# Supplementary material for: Bayesian refinement of protein structures and ensembles against SAXS data using molecular dynamics
Source: PLoS Comput Biol. 2017 Oct 18;13(10):e1005800. doi: 10.1371/journal.pcbi.1005800 (PMC5662244; doi:10.1371/journal.pcbi.1005800)
Supplement: S1 Archive — (BZ2) [file pcbi.1005800.s011.tar.bz2 › gromacs_4.62_bayesian_saxs_refi/share/html/online/g_potential.html]

g\_potential


| g\_potential Main Table of Contents |

**VERSION 4.6  
Sat 19 Jan 2013**

---

### Description

g\_potential computes the electrostatical potential across the box. The potential is
calculated by first summing the charges per slice and then integrating
twice of this charge distribution. Periodic boundaries are not taken
into account. Reference of potential is taken to be the left side of
the box. It is also possible to calculate the potential in spherical
coordinates as function of r by calculating a charge distribution in
spherical slices and twice integrating them. epsilon\_r is taken as 1,
but 2 is more appropriate in many cases.

### Files

| option | filename | type | description |
| --- | --- | --- | --- |
| **-f** | traj.xtc | Input | Trajectory: xtc trr trj gro g96 pdb cpt |
| **-n** | index.ndx | Input | Index file |
| **-s** | topol.tpr | Input | Run input file: tpr tpb tpa |
| **-o** | potential.xvg | Output | xvgr/xmgr file |
| **-oc** | charge.xvg | Output | xvgr/xmgr file |
| **-of** | field.xvg | Output | xvgr/xmgr file |

### Other options

| option | type | default | description |
| --- | --- | --- | --- |
| **-[no]h** | bool | no | Print help info and quit || **-[no]version** | bool | no | Print version info and quit || **-nice** | int | 19 | Set the nicelevel || **-b** | time | 0 | First frame (ps) to read from trajectory || **-e** | time | 0 | Last frame (ps) to read from trajectory || **-dt** | time | 0 | Only use frame when t MOD dt = first time (ps) || **-[no]w** | bool | no | View output .xvg, .xpm, .eps and .pdb files || **-xvg** | enum | xmgrace | xvg plot formatting: xmgrace, xmgr or none || **-d** | string | Z | Take the normal on the membrane in direction X, Y or Z. || **-sl** | int | 10 | Calculate potential as function of boxlength, dividing the box in this number of slices. || **-cb** | int | 0 | Discard this number of first slices of box for integration || **-ce** | int | 0 | Discard this number of last slices of box for integration || **-tz** | real | 0 | Translate all coordinates by this distance in the direction of the box || **-[no]spherical** | bool | no | Calculate spherical thingie || **-ng** | int | 1 | Number of groups to consider || **-[no]correct** | bool | no | Assume net zero charge of groups to improve accuracy |

### Known problems

- Discarding slices for integration should not be necessary.

---

http://www.gromacs.org  
gromacs@gromacs.org
